# Tobacco Smoking and Its Association with Illicit Drug Use among Young Men Aged 15-24 Years Living in Urban Slums of Bangladesh

**DOI:** 10.1371/journal.pone.0068728

**Published:** 2013-07-30

**Authors:** Mohammad Alamgir Kabir, Kim-Leng Goh, Sunny Mohammad Mostafa Kamal, Md. Mobarak Hossain Khan

**Affiliations:** 1 Department of Applied Statistics, Faculty of Economics and Administration, University of Malaya, Kuala Lumpur, Malaysia; 2 Department of Statistics, Jahangirnagar University, Savar, Dhaka, Bangladesh; 3 Department of Applied Statistics, Faculty of Economics and Administration, University of Malaya, Kuala Lumpur, Malaysia; 4 Visiting Research Fellow, Unit for the Enhancement of Academic Performance, Office of the Vice-Chancellor, University of Malaya, Kuala Lumpur, Malaysia; 5 Department of Mathematics, Islami University, Kustia, Bangladesh; 6 Department of Public Health Medicine, School of Public Health, Bielefeld University, Postfach, Bielefeld, Germany; Chancellor College, University of Malawi, Malawi

## Abstract

**Background:**

Tobacco smoking (TS) and illicit drug use (IDU) are of public health concerns especially in developing countries, including Bangladesh. This paper aims to (i) identify the determinants of TS and IDU, and (ii) examine the association of TS with IDU among young slum dwellers in Bangladesh.

**Methodology/Principal Findings:**

Data on a total of 1,576 young slum dwellers aged 15–24 years were extracted for analysis from the 2006 Urban Health Survey (UHS), which covered a nationally representative sample of 13,819 adult men aged 15–59 years from slums, non-slums and district municipalities of six administrative regions in Bangladesh. Methods used include frequency run, Chi-square test of association and multivariable logistic regression. The overall prevalence of TS in the target group was 42.3%, of which 41.4% smoked cigarettes and 3.1% smoked *bidis*. The regression model for TS showed that age, marital status, education, duration of living in slums, and those with sexually transmitted infections were significantly (p<0.001 to *p*<0.05) associated with TS. The overall prevalence of IDU was 9.1%, dominated by those who had drug injections (3.2%), and smoked *ganja* (2.8%) and *tari* (1.6%). In the regression model for IDU, the significant (p<0.01 to *p*<0.10) predictors were education, duration of living in slums, and whether infected by sexually transmitted diseases. The multivariable logistic regression (controlling for other variables) revealed significantly (*p*<0.001) higher likelihood of IDU (OR = 9.59, 95% CI = 5.81–15.82) among users of any form of TS. The likelihood of IDU increased significantly (*p*<0.001) with increased use of cigarettes.

**Conclusions/Significance:**

Certain groups of youth are more vulnerable to TS and IDU. Therefore, tobacco and drug control efforts should target these groups to reduce the consequences of risky lifestyles through information, education and communication (IEC) programs.

## Introduction

Tobacco smoking (TS) is a leading cause to many preventable and premature deaths. Recent statistics show that about 1.3 billion people smoke worldwide [1] and six million people die annually from the consequences of TS [2]. It is anticipated that by 2030, over 8 million people will die annually due to TS related health problems, of which 80% will occur in low and middle income countries [2], [3]. In addition to loss of human capital, TS can cause huge economic damage worldwide every year, especially in poor countries [2]. Unfortunately, the prevalence of TS is high among young males in low-income countries such as India (16.8%), Nepal (13.0%), Sri Lanka (12.4%), Maldives (8.5%), Pakistan (12.4%) and Myanmar (22.5%). This phenomenon may be attributed to various factors like urbanization, promotional marketing strategies of tobacco industries, westernization and misconception that associates smoking with maturity [2]. While smoking cigarettes and *bidis* are common habits among the general male population in Bangladesh, TS is also widespread among the young males (9.1%) [4]–[7].

Like TS, the prevalence of substance use and its impacts are increasingly serious. The consequences of illicit drug use (IDU) are particularly worrying in developing countries due to poor health infrastructure and limited resources to deal with the problem [8]. Geographically Bangladesh is highly vulnerable to IDU because of its proximity to the drug trafficking zones of the Golden Triangle and the Golden Crescent, and its common boundary with India (a heavy consumer of opium) and Myanmar (where drug abuse is serious) [8], [9]. TS has long been recognized as a “gateway drug” to other illicit substances, which harm both psychosocially and pharmacologically [10], [11], particularly in individuals with attention-deficit or hyperactivity disorder [12]. Numerous studies already reported that TS is strongly related to IDU in various countries [13]–[15] including Bangladesh [5], [16]–[18].

The number of urban slum dwellers has reached 1 billion, and is projected to be more than 2 billion in 2030. Rapid and unplanned urbanization along with massive rural-to-urban migration due to the combination of push and pull factors are the major forces of slum growth in developing countries including Bangladesh [19], [20]. About one-third of the urban populations in Bangladesh are slum dwellers [21], who are often neglected and deprived of basic amenities and services. Moreover, they are exposed to higher risks due to poor housing and neighborhood environment, risky lifestyles, lack of health knowledge, and poor physical and psychosocial health [17], [19]. Risky lifestyle behavior involving TS and IDU is more prevalent in slum areas [22]. As most of the slum dwellers are poor migrants from rural areas, they face difficulties in the new environment and hence suffer from poor psychosocial health linked to high stress and depression. Most of the migrants usually miss their families, friends and familiar social network and receive less support in stressful situations. High stress tends to induce smoking [23] and deviations from normal lifestyles. The health behavior model of stress indicates that individuals under stress have a higher tendency to pick up health-detrimental behaviors like TS and IDU, which is more severe among those with low income and social status [24], [25]. Lack of social networking due to anonymity in the new environment and the absence of elderly family members to provide support among new rural-to-urban migrants are also factors that lead to adoption of risky lifestyles. Undesirable features of slums increase the tendency of risky lifestyle behaviors, which is also revealed through higher prevalence of TS among slum dwellers compared to non-slum dwellers in Bangladesh [17]. Although studies on TS and IDU in Bangladesh are available [16], [17], the main contribution of this study is that it focuses on slum male youths in Bangladesh, on which information is scarce. In contrast, previous studies analyzed the adult male population [16] and examined their behavioral differences between slum and non-slum areas [17]. To the best of our knowledge, the association between TS and IDU, particularly among the young males dwelling in urban slums in Bangladesh, has not been thoroughly investigated. This study has two objectives, first, to examine the determinants of TS and IDU, and second, to investigate the association between TS and IDU among the male youths living in urban slums in Bangladesh.

We focused on young men in urban slums for various reasons: (i) the prevalence of TS and IDU is higher than that for the females [2], [8]; (ii) given the rapid urbanization process, the growth of slum populations is also on the rise [19]; (iii) slums dwellers are more vulnerable to TS and IDU because of overcrowded and stressful living conditions [17], [26]–[29]; (iv) due to nicotine addiction, younger tobacco smokers are more vulnerable to the long-term negative effects of tobacco use. Most youth smokers cannot shed their addiction as they grow into adulthood, and hence the likelihood of IDU is also higher and long-lasting among them; and (v) tobacco industries targets the youngsters as they have a longer potential time to be users and are viewed as replacement for the current smokers who quit smoking or die [2], [30].

## Data and Methods

The data were extracted from the 2006 Bangladesh Urban Health Survey (UHS). The detailed methodology of the survey including the data collection method, validation and reliability assessment is explained in the national report of the survey [31]. Briefly, the 2006 UHS employed a nationally representative sample based on a multi-stage cluster sampling approach. First, a scientifically valid sampling frame for slums (the primary sampling units) which provided the location of slum communities and their approximate populations was prepared. Based on the proportion of population in each slum to total population, the sample was selected using the probability sampling method. Next the survey set out to locate, map, and record the basic characteristics of each slum in the six City Corporations of Bangladesh. A concurrent effort involved the mapping of *mahallas* of City Corporations (along with the estimation of their population size). With this sample design, the UHS collected detailed information concerning health, health-care seeking behavior, characteristics of individuals and their households and communities in slum and non-slum areas of City Corporations, as well as the neighborhoods of District Municipalities. Given the detailed information available on the characteristics of individuals and their households and communities, the survey makes clear the categories of individuals who were most exposed to various concentrations of vulnerability. In terms of health outcomes, UHS offers a rich range of health measures, including many traditional self-reported indicators as well as more objectively measured indicators gleaned from biomarker data.

The survey gathered information of a total sample of 13,819 adult men, aged 15–59 years from slum (n = 6,488), non-slum (n = 5,667) and district municipalities (n = 1,664). From the surveyed sample, 1,576 men aged 15–24 years were from slum areas, which are the target group of this study. The National Institute of Population Research and Training (NIPORT), a research wing of the Ministry of Health and Family Welfare of Bangladesh, conducted the survey. NIPORT obtained ethical clearance from the Ministry before conducting the survey.

### The selection of variables

From the dataset, current age, marital status, level of education, religion, working status, duration of living in the slums, whether the respondent has any symptoms of STIs (sexually transmitted infections), access to television, monthly income and the wealth index which indicates wealth status were identified as independent variables for this study.

The two main dependent variables of this study are “whether the respondent is currently smoking” and “whether the respondent uses any illicit drug”. For tobacco smoking, two dichotomous questions are relevant: (i) “Do you smoke cigarette currently?” and (ii) “Do you smoke *bidi* currently?” If the response is positive, the respondents were also asked the number of cigarettes or *bidis* they smoke per day. We performed separate analyses for cigarette and *bidi* smoking because *bidis* are cheaper than cigarettes and they pose different health hazards [5]. In addition, another variable was considered by combining the two types of smoking. If a respondent smoked either cigarette or *bidi*, then he was considered as a “current smoker”, otherwise a “non-smoker”. Further, the survey recorded the use of illicit drugs such as ganja, charas, phensidle, heroin, tari, and others. If a respondent used any of the illicit drugs during the last one month prior to the survey, then he was considered as an “illicit drug user”, otherwise a “non-user”.

The details of the variables used in this study and how they were coded for analysis are presented in **Table 1**. It should be noted that the selection of variables was guided by the relevant empirical literature on TS and IDU [4], [5], [16]–[18], [32] and the health behavior model. The model attempts to explain and predict health behaviors from the attitudes and beliefs of individuals [33]. It assumes that self-destructive behavior, such as TS and IDU, occurs when individuals do not have adequate information about the health risks posed by their behavior, fail to understand their vulnerability to the consequences of their behavior, fail to understand that avoiding the behavior will reduce health risks, or encounter other informational barriers to behavior change. The theory suggests a strengthening of individuals' perception of the risk and severity of the consequences of their vulnerability might change their behavior.

**Table 1:** Variables included in the study and their coding for analysis.

Response variable: tobacco smoking (TS)		
Variables	Questions asked in the survey	Coding for analysis
Smoking cigarette, M901a	In the last 1 month, have you smoked cigarette? Options included: 1 = Yes, 2 = No	0 = Not using cigarette; 1 = Yes
Smoking *bidi*; M901b	In the last 1 month, have you smoked *bidi*? Options included: 1 = Yes, 2 = No	0 = Not using *bidi*; 1 = Yes
Prevalence of cigarette smoking (if yes), M903a	How many cigarettes do you smoke in a typical day? Option: 1 to 60 (in continuous form)	1 = 1–5 sticks daily; 2 = 6–10 sticks daily; 3 = 10+ sticks daily
Prevalence of *bidi* smoking (if yes), M905a	How many *bidis* do you smoke in a typical day? Option: 1 to 75 (in continuous form)	1 = 1–5 sticks daily; 2 = 6–10 sticks daily; 3 = 10+ sticks daily
Smoking cigarette/*bidi*; M901a, M901b	Whether respondent is currently smoking either cigarette or *bidi*. This variable is created by combining the response from M901a and M901b.	0 = No; 1 = Any one; 2 = Both cigarette and *bidi*

Response variable: illicit drug use (IDU)		
Variables	Questions asked in the survey	Coding for analysis
Use of illicit drugs: M906aa;M906ab; M906ac;M906ad; M906ae;M907aa; M907ab	In the last 1 month, have you used the following? Options: Ganja (1 = yes, 2 = no); Charas (1 = yes, 2 = no); Phensidle (1 = yes, 2 = no); Heroin (1 = yes, 2 = no); Tari (1 = yes, 2 = no); Pethedine (1 = yes, 2 = no); Morphin (1 = yes, 2 = no)	0 = No, for all cases ; 1 = Yes, any one of the mentioned drugs

Predictors/independent variables		
Variables	Questions asked in the survey	Coding for analysis
Age in years, M101A	How old are you at your last birthday? (in continuous form)	1 = 15–19; 2 = 20–24
Marital status, M102	Marital status? 1 = Currently married; 2–5 = separated/deserted/widowed/divorced; 6 = never married	1 = Ever married (1–5)#; 2 = Never married (only 6)
Education, M104A	What level of schooling have you last attended? (in open form)	0 = No education; 1 = Primary; 2 = Secondary; 3 = Higher*
Religion, M109	What is your religion? 1 = Islam; 2 = Hinduism; 3 = Buddhism; 4 = Christianity; 6 = Others	1 = Islam; 2 = Others (2–6)
Currently working, M115	Are you currently working? Option: 1 = Yes; 2 = No	1 = Yes; 2 = No
Duration in slums, M127	How long have you been here? Option: 1–24 years (in continuous form); 95 = Less than 1 year; 97 = Always	1 = <5 years; 2 = 5–9 years; 3 = 10-<24 years; 4 = Permanent
Have any STIs? M503AA, M503AB, M503AC	Sexually Transmitted Diseases, any discharge from penis? (Option: yes, no), any sore on or near penis? (Option: yes, no), any pain during urination? (Option: yes, no)	0 = No, for all options; 1 = Yes, for any of the options
TV Watching, M108	Do you watch TV? 1 = Yes, 2 = No	1 = Yes; 2 = No
Income, M120	Net salary/wage during the last month in (BDT)¶ (in continuous form)	0 = No income (None); 1 = <5000; 2 = 5000+
Wealth quintiles**	Basic durable goods and assets in household	1 = poorest; 2 = poor; 3 = middle; 4 = rich; 5 = richest

**Note:**

# currently married or married at least once;

* at least 11 years of education;

¶ Bangladeshi Taka and exchange rate is 78.11 BDT/USD;

** quintiles based on principal component analysis.

### Statistical analyses

Statistical analyses were performed using SPSS version 18 (SPSS Inc, Chicago, IL). Frequency runs were generated to compute the prevalence of TS and IDU. Bivariable analyses using cross tabulations were performed to obtain the prevalence of TS and IDU for various categories of the selected variables and to identify significant determinants using the Pearson's Chi-square (χ^2^) test [34]. The null hypothesis of no relationship between tobacco smoking (illicit drug use) and the independent variable is rejected if the p-value of the test statistic is less than 0.05 (P<0.05). Determinants that significantly explain both TS and IDU were entered into the logistic regressions for multivariable analyses [35]. We utilized two binary logistic regression models separately, first for cigarette or *bidi* smoking and second for the use of any illicit drug. In both cases, a multivariable binary logistic regression analysis was conducted to examine the significance of the influencing factors of TS and IDU.

The logistic regression model is given by:
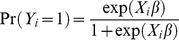



Where 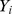 is a binary variable that takes a value of ‘
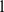
’ if the respondent is a current smoker (illicit drug user) and ‘
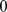
’ otherwise, 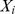 is a vector of independent variables and 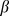 is a vector of unknown parameters.

The estimated form of the general logistic transformation can be expressed as
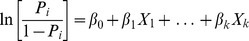
(1)


The odds ratio (OR) in favor of 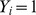 together with its 95% confidence interval (CI) were computed for 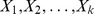 to indicate how many times the group of interest is more likely to be a smoker (illicit drug user) compared to the reference group.

Another model of multivariable binary logistic regression was also estimated to examine the association between TS and IDU after controlling for socio-economic and demographic background and the model is:
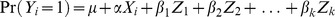
(2)Where 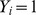 if respondent-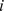 is an illicit drug user and 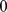 otherwise, 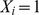 if respondent-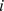 is a smoker and 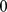 otherwise, and 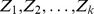 are variables representing the background characteristics that affect IDU. For instance, 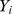 was assigned a value of 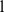 if the respondent had taken any IDU. The TS variable 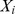 was assigned a value of 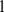 if the respondent were a current smoker. For comparison purposes, the regression was also estimated separately for the two different forms of smoked tobacco products, namely, cigarettes and *bidis*. The odds ratios and their 95% confidence interval for examining the impact of smoking on IDU after controlling for socio-economic characteristics were compared.

## Results

### Profile of the respondents

Basic information of the respondents is provided in **Table 2**. More than 70% of the respondents were in the age group of 20–24 years, and about 30% of them aged 15–19 years. Less than one third (31%) of the respondents were ever married (currently married, divorced or widowed). Most of the respondents attained either primary (33%) or secondary (40%) education. About one-fifth (18%) did not have any formal education, whereas 10% had post-secondary education. More than 95% of the respondents were Muslims. Majority of these youths (88%) were currently working. About two-fifth (41%) had resided in the slum areas permanently while only 22% were living in slums for less than 5 years. Slightly over 4% of the male youths in the slums reported symptoms of STIs. Almost all (95%) had access to television. Some 30% of the youths had no income and 65% had a monthly income of less than BDT5000. In terms of wealth index, more than 58% were from the bottom 40% poorest groups while only 19% were from the top 40% richest groups.

**Table 2:** Socio-demographic profile of young men living in urban slums in Bangladesh, UHS 2006.

Characteristics	N	%
**Age in years**		
15–19	454	28.8
20–24	1122	71.2
**Marital status**		
Ever married#	483	30.6
Never married	1093	69.4
**Level of education**		
No education	276	17.5
Primary	513	32.5
Secondary	625	39.6
Higher*	162	10.3
**Religion**		
Islam	1500	95.2
Others	75	4.8
**Currently working**		
No	185	11.7
Yes	1391	88.3
**Duration in slums (in years)**		
<5	353	22.4
5–9	304	19.3
10-<24	273	17.4
Permanent	645	41.0
**Have any STIs?**		
No	1508	95.7
Yes	68	4.3
**TV Watching**		
No	84	5.3
Yes	1492	94.7
**Income per month (BDT)** ¶		
None	478	30.3
<5000	1021	64.8
5000+	76	4.8
**Wealth index/quintiles** **		
Poorest	497	31.6
Poor	419	26.6
Middle	351	22.3
Rich	226	14.3
Richest	82	5.2
Total	1576	100.0

**Note:**

# currently married or married at least once;

* at least 11 years of education;

¶ Bangladeshi Taka and exchange rate is 78.11 BDT/USD;

** quintiles based on principal component analysis.

### Prevalence of TS and IDU

The current smoking prevalence among the respondents was 42.3%, with the rate of smoking cigarettes at 41.4% and *bidis* at 3.1% (**Table 3**). The average daily consumption of cigarettes and *bidis* were about 8 and 11 sticks respectively. Of those smoking, about 60% of the young male slum dwellers smoked at least 6 sticks of cigarettes daily. Close to one fifth (18.6%) of them smoked an average of 18 cigarettes per day. Some 56% of the *bidi* users consumed at least 6 sticks per day, and 30% of them had an average daily intake of 23 sticks. About 9.1% of the youths were involved in IDU. The main source of drug abuse was injectable drugs (3.2%). The other more serious cases involved the use of ganja (2.8%) and tari (1.6%).

**Table 3:** Tobacco and illicit drug use among the young men living in urban slums in Bangladesh, UHS 2006.

Tobacco/Drug Use	N	% currently smoking	Mean ± Standard deviation
**Smoking cigarette** #			
No	923	58.6	—
Yes	653	41.4	8.3±5.7
If yes			
1–5 per day	264	40.4	3.7±1.3
6–10 per day	268	41.0	8.5±1.6
10+ per day	121	18.6	18.2±5.0
**Smoking ** ***bidi*** #			
No	1527	96.9	—
Yes	49	3.1	10.9±10.5
If yes			
1–5 per day	22	44.4	3.8±1.4
6–10 per day	12	25.3	8.8±1.7
10+ per day	15	30.2	23.2±12.5
**Smoking cigarette/** ***bidi*** #			
No	910	57.7	—
Any one	630	40.0	—
Both	36	2.3	—
**Illicit drugs (IDs) taken in last one month before the survey** ¶			
Ganja (marijuana)	44	2.8	—
Phensidle*	6	0.4	—
Heroin	3	0.2	—
Tari**	15	1.6	—
Injected any drugs***	51	3.2	—
Others‡	24	0.9	—
Total	143	9.1	—

**Note:**

# total respondents for tobacco smoking is 1576 and for.

¶ IDU is 143;

* a cough syrup containing codeine;

** locally made palm wine;

*** injected drugs mainly pethedine, or morphine;

‡ charas (hashis).

### Factors associated with TS and IDU

The variables that were significantly (*p*<0.001) associated with cigarette and *bidi* smoking among young men in urban slums include age, marital status, education, current working status, whether they have symptoms of any STIs, and wealth index (**Table 4**). Although duration of living in slums was not associated with *bidi* smoking, it has a significant impact (P<0.001) on those who smoked cigarettes. Those who dwelled in the slums for a longer period had a higher tendency to be cigarette users. Income was significantly associated (P<0.001) with only *bidi* smoking but did not affect the behavior of cigarette smokers. The significant determinants of IDU include age, marital status, education, duration of stay in slums, and whether the respondents had symptoms of STIs (P<0.001). As is the case for TS, those with better education attainment were less likely to be involved in IDU. The prevalence of IDU was higher among the migrants who stayed in the slums for a longer period and those with symptoms of STIs. It must also be noted that IDU was highly associated (P<0.001) with TS. Notably, the prevalence of IDU was higher among those who were heavy cigarette smokers.

**Table 4:** Prevalence of TS and IDU among the young men living in urban slums in Bangladesh, UHS 2006.

Characteristics	Currently smoking (% yes)			(% yes)
	Cigarettes	*Bidis*	Cigarettes/*bidis*	IDU
**Age in years**	11.6; P<0.001	3.8; P<0.0001	14.3; P<0.001	3.9; P<0.001
15–19	34.8	1.7	34.9	6.8
20–24	44.1	3.7	45.2	10.0
**Marital status**	58.4; P<0.001	32.1; P<0.001	67.4; P<0.001	10.7; P<0.001
Ever married	55.7	6.8	57.6	12.6
Never married	35.1	1.5	35.5	7.5
**Level of education**	78.0; P<0.001	53.2; P<0.001	91.0; P<0.001	15.8; P<0.001
No education	59.9	9.7	63.1	13.6
Primary	44.9	2.8	45.4	10.2
Secondary	35.7	1.3	36.0	7.7
Higher	21.2	.0	21.2	2.8
**Religion**	0.9; P = 0.328	0.05; P = 0.820	0.77; P = 0.380	0.10; P = 0.75
Islam	41.7	3.1	42.5	9.1
Others	36.3	2.9	37.8	8.5
**Currently working?**	10.8; P<0.001	6.7; P<0.001	12.4; P<0.001	2.4; P = 0.12
No	30.2	0.00	30.2	6.1
Yes	42.9	3.5	43.9	9.5
**Duration in slums (years)**	12.8; P<0.01	1.8; P = 0.609	13.1; P<0.001	24.9; P<0.001
<5	33.3	2.5	34.2	4.2
5–9	44.4	4.3	46.2	7.3
10-<24	44.6	2.9	46.1	15.5
Permanent	43.2	2.9	43.2	9.9
**Have any STIs?**	7.4; P<0.001	7.9; P<0.001	9.5; P<0.001	6.6; P<0.001
No	40.7	2.8	41.5	8.7
Yes	57.3	9.1	60.1	17.8
**TV Watching**	1.4; P = 0.237	0.1; P = 0.802	1.05; P = 0.307	1.0; P = 0.32
No	47.6	3.5	47.6	6.3
Yes	41.1	3.1	42.0	9.2
**Income per month (BDT)**	2.3; P = 0.326	13.9; P<0.001	3.1; P = 0.212	0.7; P = 0.70
None	39.7	0.9	39.8	9.3
<5000	41.7	4.3	42.8	8.8
5000+	48.8	1.6	50.3	11.2
**Wealth index**	20.2; P<0.001	27.5; P<0.001	26.0; P<0.001	4.8; P = 0.309
Poorest	46.1	6.2	48.7	9.7
Poorer	46.2	1.0	46.2	10.5
Middle	35.4	3.0	35.4	6.1
Richer	35.7	1.5	35.7	9.8
Richest	30.4	.0	30.4	8.3
**Smoking cigarette/** ***bidi***	—	—	—	127.2; P<0.001
No	—	—	—	2.1
Yes	—	—	—	18.5
**No. of cigarettes per day**	—	—	—	148.3; P<0.001
None	—	—	—	3.8
1–5	—	—	—	9.6
6–10	—	—	—	20.7
10+	—	—	—	33.9
**No. of ** ***bidis*** ** per day**	—	—	—	58.2; P<0.001
None	—	—	—	8.2
1–5	—	—	—	32.7
6–10	—	—	—	60.3
10+	—	—	—	27.1
**Total**	41.4	3.1	42.3	9.1

**Note:** Figures in the first row of every independent variable are the chi-squared statistics and p-values for the tests of association.

### Multivariable regression results

The results of multivariable logistic regression (**Table 5**) of TS and IDU revealed that the older males were more likely (OR = 1.32; 95% CI = 1.04–1.69) to smoke tobacco than their younger counterparts. Youths who were ever married had almost two times higher likelihood of TS than those who were not married. Illiteracy was associated with five-time higher likelihood of TS than those with at least 11 years of education. The odds ratio reduced to 2.7 for those with primary education and 1.95 for secondary education. All the migrants to the slum areas were 1.4–1.6 times more likely to be smoker compared to those who stayed in the slums permanently. The male youths who reported having STI symptoms were at increased risk (OR = 2.13, 95% CI = 1.27–3.57) of TS.

**Table 5:** Multivariable logistic regression analysis of TS and IDU by background characteristics of the young men living in urban slums in Bangladesh, UHS 2006.

Characteristics	Smoking cigarette/*bidi*		Use of any illicit drug	
	OR	95% CI	OR	95% CI
**Age in years**				
15–19	1.00	—	Ns	—
20–24	1.32^b^	1.04–1.69	Ns	—
**Marital status**				
Ever married	1.92^a^	1.51–2.44	Ns	—
Never married	1.00	—	Ns	—
**Level of education**				
No education	5.00^a^	3.13–7.98	2.28^d^	0.82–6.39
Primary	2.71^a^	1.76–4.15	2.42^c^	0.89–6.61
Secondary	1.95^a^	1.29–2.97	2.19^d^	0.80–5.97
Higher	1.00	—	1.00	—
**Duration in slums (years)**				
<5	1.45^c^	1.04–2.01	0.44^b^	0.24–0.80
5–9	1.27^c^	0.90–1.79	0.61^c^	0.36–1.03
10-<24	1.62^a^	1.22–2.15	1.58^c^	1.01–2.48
Permanent	1.00	—	1.00	—
**Have any STIs?**				
No	1.00	—	1.00	—
Yes	2.13^b^	1.27–3.57	1.74^d^	0.87–3.50

**Note:** **OR** – odds ratio; **CI** – confidence interval;

a P<0.001;

b P<0.01;

c P<0.05; and

d P<0.10; Ns = Not significant.

Those with less education were at least twice more likely to use illicit drugs compared to the youths with at least 11 years of education. However, the difference between the three categories of education (no education, primary and secondary) was not as clear as for TS. While the migrants who stayed in the slums for less than 10 years had a lower tendency of IDU than the youths who lived there permanently, the highest likelihood of IDC was among the migrants who lived there for at least 10 years. Tendency to be illicit drug users was also higher among those who had symptoms of STIs.

**Figure 1:**
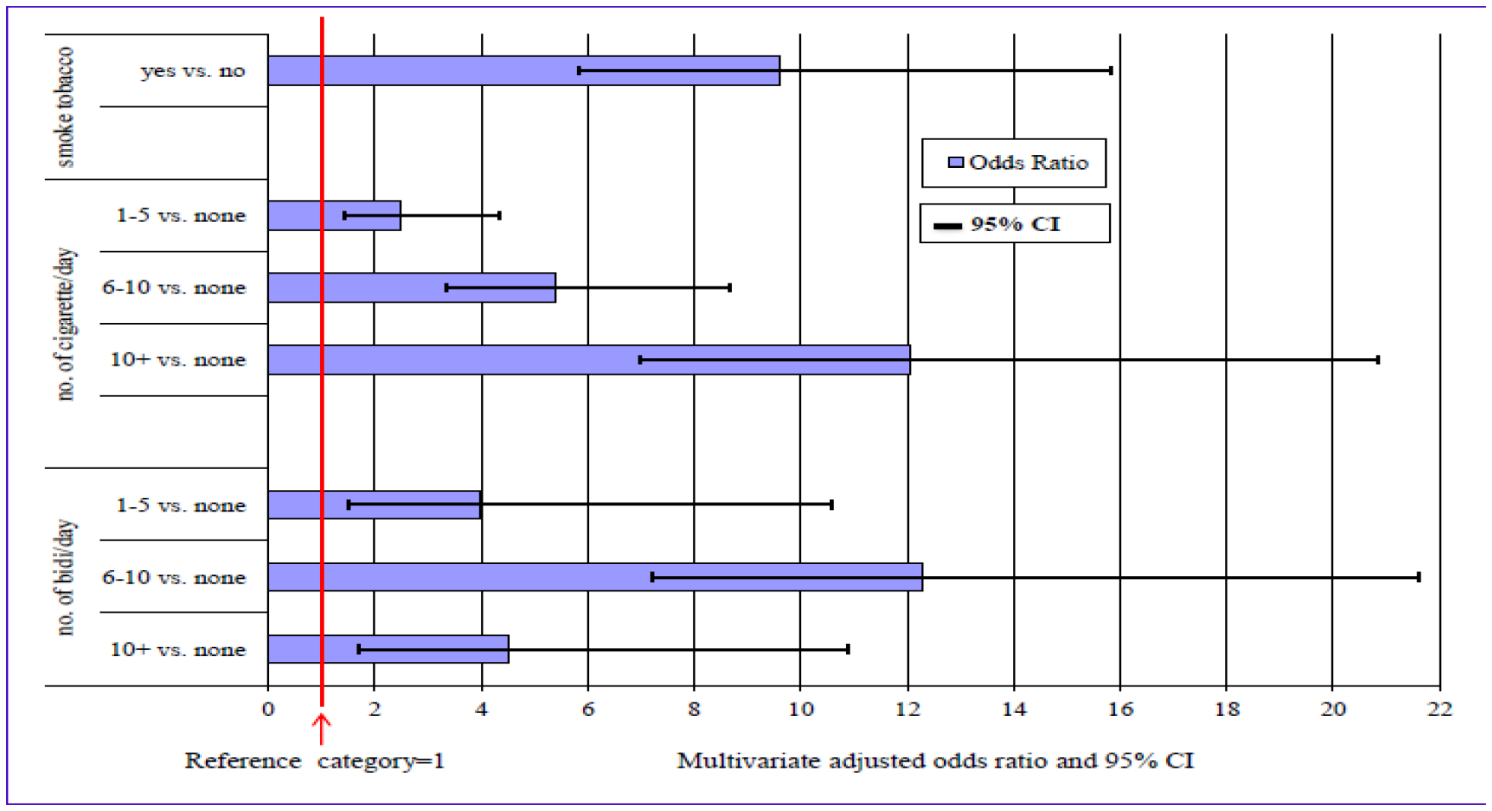
OR and 95% CI of illicit drug users to tobacco smokers adjusted for confounders.

Further analyses were performed to assess the impact of smoking on IDU by estimating the logistic regression for the latter controlling for the confounding factors of age, marital status, education, duration of living in slums, and having symptoms of STIs. These factors are determinants found to be significant earlier in **Table 4**. The odds ratios and their 95% CI for IDU in relation to TS are plotted in **Figure 1**. The results indicated that the respondents who smoked tobacco (cigarettes or *bidis*) revealed 9.6 times higher likelihood of using any illicit drugs compared to the non-tobacco users.

The odds ratio increased with the number of cigarettes smoked daily. Those who smoked 5 cigarettes or less per day were between two to three times more likely to use illicit drugs compared to non-tobacco users, but the likelihood for those who smoked 10 or more cigarettes per day increased to 12 times higher. The risk of IDU for *bidi* smokers could be up to 12 times higher than the youths who do not smoke *bidis* depending on the amount of daily *bidi* consumption.

## Discussion

The study revealed that about two-fifth (42.3%) of the young men aged 15–24 living in urban slums of Bangladesh were tobacco smokers. This prevalence is much higher when compared with youths of the population in Bangladesh (9.1%) and other neighboring countries such as, India (16.8%), Nepal (13.0%), Sri Lanka (12.4%), Maldives (8.5%), Pakistan (12.4%) and Myanmar (22.5%) [2]. It was also found that the male youths in slums are more likely to use cigarettes than *bidis*, which could be associated with urban culture, working status and availability of tobacco products [17]. Like the higher prevalence of TS among urban slum youths, the rate of IDU (9.1%) was also 2.6 times higher than the rate (3.4%) for the youths in Bangladesh [5], [8], [16], [36]. A higher level of risky behaviors in deprived or overcrowded areas was also reported in other studies [17], [26]–[29]. Unhealthy lifestyles in adverse socio-economic conditions, weak social norms and cultural beliefs, undesirable neighborhood characters, availability of tobacco products, and lack of preventive services in the deprived areas may have significant influence on individuals' behaviors [27], [28], [37].

Several demographic, socioeconomic and behavioral factors are identified as significant determinants of both TS and IDU among young men in urban slums. For instance, among the youths, significantly higher prevalence of TS and IDU were found among those aged 20–24 years, which are consistent with the findings of other studies in Bangladesh and elsewhere [5], [16], [17], [38]–[40]. This may be partially attributed to the traditional and cultural norms in Bangladesh and stress related issues. In the prevailing cultural norms of the Bangladeshi society, TS among youths is discouraged by elders and family members. TS by any younger members in the presence of older people are viewed as indecent and intolerable. IDU is totally prohibited in Bangladesh. In line with other studies [5], [41], [42], TS and IDU revealed a strong inverse association with level of education. The likelihoods of TS and IDU were found to be approximately five and two times higher among male youths with no formal education, respectively, compared to those with post-secondary education. The finding suggests that improvement of education could be an important strategy for reducing both TS and IDU in urban slums. The health behavior model of stress indicates that populations under stress generally engage in health detrimental behaviors, particularly in the context of low social status [43]. Based on this model, we could say that stressful conditions in slums [17] may lead to an increased risk of smoking and illicit drug use [23]–[25], [44], which is also evident from the higher prevalence of such risky behaviors among the slum youths compared to the youths in the country. Consistent with the health behavior model of stress [43] and other empirical studies [17], the likelihood of TS among youths in new settlements (like slums) is significantly (P<0.001) higher than the youths who lived in the same places since birth. While the likelihood of IDU is higher among the young male migrants who had settled in the slums for a long period of time, the likelihood among the recent migrants (duration less than 10 years) is lower. This may be partly attributed to the social network and environmental factors. Similarly, the higher rates of TS and IDU among the youths having symptoms of STIs could be outcomes of their risky lifestyles in poorly managed living conditions [5], [16], [38].

This study found a significant (P<0.001) positive association between TS and IDU which was consistent with other studies [16], [18], [45], [46]. Other empirical evidence also supported the relationship between TS and IDU. For example, regular use of tobacco was the predictor of life-time drug use [47], [48]. Our study showed that the likelihood of IDU was more than 9 times higher among slum youths who smoked regularly. In line with other studies [5], [16], [18], [49], the likelihood of IDU of any form increased with more frequent cigarette smoking. Although the likelihood of IDU increased with *bidi* smoking, but its relationship with the consumption rate of *bidis* is not as clear as that for cigarette smoking.

The strong association between TS and IDU should not be ignored in policy designs and interventions. Cross-sectional data used here are not suitable for making inference on ‘cause-effect’ relationship. However, according to the discussion of [16], generally the smoking behavior is first picked up and then followed by IDU. As both are of public health concerns, their consequences could be reduced by employing suitable intervention programs at different levels. Government and non-government organizations, community and family should be engaged in intervention programs and health promotional activities. Considering the consequences of TS, the government of Bangladesh announced several strategies for tobacco control in the National Plan of Action ‘2007–2010’. These strategies include setting appropriate price and tax policies; prohibition of advertisements and sponsorship; raising awareness through training, education and communication; restrictions on sales of tobacco products to minors; and labeling the harmful effects of tobacco products on packaging [50]. Some of these strategies are already implemented. Now, advertisements of cigarettes and *bidis* will have to include a warning message stating that smoking is hazardous for health. Warning messages are obligatory on the packaging of cigarettes and *bidis*. Regrettably, these printed messages on packaging are not totally effective for slum youths as many of them are illiterate. Moreover, as many smokers in urban slums buy cigarettes or *bidis* by the sticks rather than full packets, they miss the warning labels on the packaging [4], [17].

In connection with other policies [4], [17], [50], some general recommendations emerge from this study. First, given the importance of awareness, digital posters carrying warning messages on the adverse effects of TS and its relation with IDU can be displayed at places in slums where youths gather and also at points of sale. Second, community leaders along with NGO activists and law enforcing agencies should act jointly to reduce the number of available points for sale of tobacco products. They should also restrict smoking to specific places to reduce exposure of other youths to second hand smoking and prevent them from emulating the behavior. Third, since the majority of youths are Muslims, tobacco control strategies should involve religious leaders especially *Imams* (head of a mosque). They may deliver brief messages about the harmful effects of TS and IDU during Friday prayers (performed by the Muslims together in mosques on Fridays) as a part of tobacco and drug control policy. Fourth, as more than 50% of the youths are poor, they tend to be more price sensitive [51], [52], and increasing prices of tobacco products may limit their usage. Another recommendation is that parents, teachers, elders and other respected persons in the society should assist to prevent the youths from adopting health risky behaviors through close monitoring and mentoring.

The findings in this paper are subject to a few limitations. Self-reported data on TS and IDU from Bangladesh Urban Health Survey could suffer from recall bias and deliberate misreporting. Even though anonymity and confidentiality were ensured during the survey, respondents might have under-reported the incidence as TS and IDU in Bangladesh are not widely acceptable social norms. Such misreporting could under estimate both the prevalence of TS and IDU and lead to inaccurate measures of their relationship with other variables. Further, STIs may be under reported as the negative social stigma associated with STIs will put some pressure on the respondents in revealing the truth. Therefore, the behavioral factors explored in this study are preliminary. Furthermore, the survey did not include information on tobacco and drug initiation and cessation along with TS and IDU history of family members, and familial or environmental stress that may substantially influence smoking and drug abuse behavior. Although many available variables were analyzed, exclusion of some other important variables at the individual, family and community level such as parental background, social networking, sexual behavior and urbanization might limit our findings. A qualitative study is suggested to supplement the understanding of the determinants of TS and IDU among youths in urban slums.

Despite these limitations, the current study provides useful contributions. According to our findings, the prevalence of TS and IDU were remarkably higher among slum male youths compared to the population in general. Although our findings were not directly comparable, some studies found higher prevalence of TS among migrants than non-migrants [53], [54]. Based on these studies, we argue that higher prevalence of TS and IDU among slum youths may be related to rural-to-urban migration. Generally migrants are distinct and unprivileged group in cities, who are mainly employed in low-paying and hazardous jobs [54]. Migration may disrupt social support and network system and the migrants face a higher level of stress as they need to cope with new living conditions, social and cultural contexts and intense competitions [53]. Briefly, isolation from home and the lack of social support, pace of city life along with unstable living and employment conditions may induce a high level of stress among migrants, which ultimately increases the likelihood of smoking [53], [54], [55]. Some smokers also perceive smoking as a coping strategy to reduce stress, anxiety, sadness and anger [55]. Slums in rapidly urbanizing countries are generally featured by poor housing, overcrowding, poor environmental and healthcare services, and other risk factors related to unhealthy lifestyles [20], [56]. In Bangladesh, about 40% of the urban populations are slum dwellers, mostly migrants from rural areas. Rural-based push factors as well as urban-based pull factors lead to migration to urban areas particularly among the youths and adults [56]. The uncontrolled growth of slums put enormous strains on the urban infrastructure and environmental sustainability, thereby influencing the health of slum population in general and slum male youths in particular [20], [53], [56]. In conclusion, TS among young men living in urban slums is high compared to other youths in Bangladesh and other neighboring countries. Moreover, TS was found to be positively associated with IDU. Of all the predictors of IDU, TS revealed the strongest association with IDU. Since both tobacco and illicit drugs are perilous in all aspects and young people from poor families in slums are more likely to be vulnerable, comprehensive strategies combining the proposed and existing policies should be implemented to overcome these problems.
