# Correction: Tobacco Smoking and Its Association with Illicit Drug Use among Young Men Aged 15-24 Years Living in Urban Slums of Bangladesh

**DOI:** 10.1371/journal.pone.0091618

**Published:** 2014-02-28

**Authors:** The PLOS ONE Staff

Affiliation number 5 for the third author, Sunny Mohammad Mostafa Kamal, is incorrect. The correct affiliation is: Department of Mathematics, Islamic University, Kushtia, Bangladesh.
